# Prevention of congenital malformations and other adverse pregnancy outcomes with 4.0 mg of folic acid: community-based randomized clinical trial in Italy and the Netherlands

**DOI:** 10.1186/1471-2393-14-166

**Published:** 2014-05-13

**Authors:** Renata Bortolus, Fenneke Blom, Francesca Filippini, Mireille NM van Poppel, Emanuele Leoncini, Denhard J de Smit, Pier Paolo Benetollo, Martina C Cornel, Hermien EK de Walle, Pierpaolo Mastroiacovo

**Affiliations:** 1 Office for Research Promotion, Department of the Hospital Management and Pharmacy, Verona University Hospital, P.le A. Stefani, 1-37126 Verona, Italy; 2 Community Genetics, Department of Clinical Genetics, EMGO Institute for Health and Care Research, VU University Medical Center, Amsterdam, the Netherlands; 3 Department of Public and Occupational Health, EMGO Institute for Health and Care Research, VU University Medical Center, Amsterdam, the Netherlands; 4 Alessandra Lisi International Centre on Birth Defects and Prematurity-ICBD, WHO Collaborating Centre, Rome, Italy; 5 MediClara Projects BV, Abcoude, the Netherlands; 6 Healthcare Directorate, Verona University Hospital, Verona, Italy; 7 Department of Genetics, University Medical Center Groningen, University of Groningen, Groningen, the Netherlands

**Keywords:** Congenital malformations, Birth defects, Folic acid, Vitamins, Prevention, Prematurity, Birth weight, Pre-eclampsia, Meta-analysis

## Abstract

**Background:**

In 2010 a Cochrane review confirmed that folic acid (FA) supplementation prevents the first- and second-time occurrence of neural tube defects (NTDs). At present some evidence from observational studies supports the hypothesis that FA supplementation can reduce the risk of all congenital malformations (CMs) or the risk of a specific and selected group of them, namely cardiac defects and oral clefts. Furthermore, the effects on the prevention of prematurity, foetal growth retardation and pre-eclampsia are unclear.

Although the most common recommendation is to take 0.4 mg/day, the problem of the most appropriate dose of FA is still open.

The aim of this project is to assess the effect a higher dose of peri-conceptional FA supplementation on reducing the occurrence of all CMs. Other aims include the promotion of pre-conceptional counselling, comparing rates of selected CMs, miscarriage, pre-eclampsia, preterm birth, small for gestational age, abruptio placentae.

**Methods/Design:**

This project is a joint effort by research groups in Italy and the Netherlands. Women of childbearing age, who intend to become pregnant within 12 months are eligible for the studies. Women are randomly assigned to receive 4 mg of FA (treatment in study) or 0.4 mg of FA (referent treatment) daily. Information on pregnancy outcomes are derived from women-and-physician information.

We foresee to analyze the data considering all the adverse outcomes of pregnancy taken together in a global end point (e.g.: CMs, miscarriage, pre-eclampsia, preterm birth, small for gestational age). A total of about 1,000 pregnancies need to be evaluated to detect an absolute reduction of the frequency of 8%. Since the sample size needed for studying outcomes separately is large, this project also promotes an international prospective meta-analysis.

**Discussion:**

The rationale of these randomized clinical trials (RCTs) is the hypothesis that a higher intake of FA is related to a higher risk reduction of NTDs, other CMs and other adverse pregnancy outcomes. Our hope is that these trials will act as catalysers, and lead to other large RCTs studying the effects of this supplementation on CMs and other infant and maternal outcomes.

**Trial registration:**

Italian trial: ClinicalTrials.gov Identifier: NCT01244347.

Dutch trial: Dutch Trial Register ID: NTR3161.

## Background

### Congenital malformations and other adverse outcomes

Congenital malformations (CMs) are an important public health issue in terms of impact on the quality of life of affected children, adults and their families, and are a major cause of early spontaneous abortions, termination of pregnancy, infant morbidity, mortality and disability in all industrialized countries. Even in the less developed countries, CMs are recognized as one of the major problems in the maternal-child health field.

The European Surveillance of Congenital Anomalies (EUROCAT), a network of population-based congenital anomaly registries in Europe, currently surveys more than 1.7 million births per year, including 31% of births in the European Union. EUROCAT member registries collect data, ascertained from multiple sources, on all major structural congenital and chromosomal anomalies: the prevalence rate of all anomalies, excluding chromosomal anomalies, is 219.70 per 10000 births, and the rate of neural tube defects (NTDs: anencephalus and similar, spina bifida, encephalocele) is 9.32 per 10000 births (2007–2011). Across Europe, an estimated 4500 pregnancies are affected by NTDs each year [1].

The economic cost of CMs is very high, including long-term psychological and socio-economic effects. In particular, the lifetime direct medical cost for patients with NTDs is significant, with the majority of cost being for inpatient care, for treatment at initial diagnosis in childhood, and for comorbidities in adult life. Caregiver time costs are also significant [2]. The lifetime indirect cost for patients with spina bifida is even greater due to increased morbidity and premature mortality [3].

Considering other adverse pregnancy outcomes, preterm birth is the second largest direct cause of child deaths in children younger than 5 years and preterm birth complications are estimated to be responsible for 35% of the world’s 3.1 million annual neonatal deaths. Available evidence suggests that the rate of preterm birth in high-income countries is rising [4]. The majority of this increase is due to a rise in the rate of late preterm births (between 34 and 36 weeks gestation). Preterm birth, defined as delivery prior to 37 completed weeks, is a major challenge for maternal and perinatal care worldwide and a leading cause of neonatal morbidity and mortality [4-6]. In 2010, an estimated 14.9 million babies (range 12.3-18.1 million) were born preterm; this is 11.1% of all live births worldwide, ranging from about 5% in several European countries to 18% in some African countries. Children born prematurely have higher rates of learning disabilities, cerebral palsy, sensory deficits and respiratory illnesses compared to children born at term. These negative health and developmental effects often extend to later life, resulting in enormous medical, educational, psychological and social costs [6-8].

Birth weight is the endpoint of different foetal growth patterns and adverse exposures that may influence foetal growth as early as the first trimester. Human growth and development rates are highest during the first trimester of pregnancy, when essential foetal organ development is completed. Foetal developmental adaptations due to adverse conditions may affect the structure, physiology and function of various organ systems, leading to foetal growth restriction and increased risks of metabolic and cardiovascular disease in adulthood. Foetal growth restriction is widely recognized as a risk factor for neonatal mortality and morbidity [9], as well as reduced cognitive function and the development of chronic diseases in later life [6,10,11].

Regarding maternal adverse outcomes, pre-eclampsia is a major cause of maternal mortality (15-20% in developed countries) and morbidities (acute and long-term), perinatal deaths, preterm birth and intrauterine growth restriction [12,13]. Despite advances in perinatal care, frequency of pre-eclampsia has not changed. Several studies have also suggested that women who develop pre-eclampsia are at increased risk of cardiovascular complications later in life [14-16], in particular hypertension, ischaemic heart disease, stroke, venous thromboembolism, and death [17].

Primary prevention of these adverse pregnancy outcomes in the population is a crucial policy priority, including pre-conceptional care and whole population approaches [1,7]. Furthermore, finding simple and inexpensive ways to prevent these infant and maternal adverse outcomes is salient in any setting [2,18,19].

### Folic acid supplementation

Folic acid (FA) (vitamin B9 or pteroyl-L-glutamic acid) is a water-soluble vitamin of the B group. It is a synthetic product not available in nature, and represents the main compound in multivitamin products and in cereals and wheat fortification. The 5-metyl-tetrahydrofolate (5-metilTHF) is the form with greater biologic activity, and the FA must be transformed into this molecule in order to exert its biological function. Folates act as cofactors of enzymes involved in the aminoacid metabolism, in the DNA and RNA synthesis (purine and pyrimidine synthesis) [20] and, together with vitamin B12, in the methylation of nucleic acids, proteins and lipids [21-23]. A central feature of foetal development is widespread and sustained cell division. As a result of its role in nucleic acid synthesis, the need for folate increases during times of rapid tissue growth [24]. From the point of view of preventive medicine and clinical pathology, the study of the interaction of folate metabolism and that of homocysteine/methionine is particularly promising [25,26].

Folate insufficiency can be due to inadequate dietary intake, utilization defect, typically in alcohol abuse and hepatic diseases, malabsorption, increased needs like in pregnancy and during lactation, interference by drugs (anti-convulsants, chloramphenicol, methotrexate, oral contraceptives, pyrimethamine, sulfasalazine and other sulphamidics, trimethoprim), smoking, deficiency of enzymes and cofactors necessary to the generation of active forms of FA.

Moreover, the folate functional insufficiency is emerging as one of the most common nutritional deficiencies. The first consequence of even light folate insufficiency is an increase of plasmatic homocysteine. Such a condition is clinically silent, but is now recognized as a metabolic risk factor for many multifactorial diseases [27,28].

The association between folate insufficiency and congenital defects has been intensely studied in the past twenty years. In the context of foetus development disorders, the NTDs represent the paradigm of the CMs avoidable by means of FA intake.

In 1991, in a randomized clinical trial (RCT) performed in the 1983–1991 years, the Medical Research Council Vitamin Study Group [29] showed that 4.0 mg of peri-conceptional FA supplementation reduced the risk of recurrence of NTDs by 72% (RR = 0.28, 95% CI 0.12-0.71); in 1992 the Hungarian RCT [30], performed in the period 1984–1991, showed that supplementation with multivitamins including 0.8 mg of FA reduced the risk of the occurrence of the same condition (OR = 0.07, 95% CI 0.04-0.13). These two RCTs have shown conclusively that the risk of NTDs can be reduced substantially by taking FA during the peri-conceptional period (at least 1 month before the last menstrual period up to the end of the first trimester of pregnancy). Previous and subsequent investigations confirm these results [31,32].

In 2010 a Cochrane review [33] confirmed that FA supplementation prevents the first- and second-time occurrence of NTDs. Five trials involving 6105 women (1949 with a history of a pregnancy affected by an NTD and 4156 with no history of NTDs) were included. Overall, the results were consistent in showing a protective effect of daily FA supplementation (alone or in combination with other vitamins and minerals) in preventing NTDs compared with no interventions/placebo or vitamins and minerals without FA (RR = 0.28, 95% CI 0.15-0.52). FA had a significant protective effect for recurrence (RR = 0.32, 95% CI 0.17-0.60). There were insufficient data to evaluate the effects on other outcomes such as cleft lip and palate, congenital cardiovascular defects, or any other birth defects.

However, the Hungarian RCT [30] and some observational studies [34-37] suggest that FA may reduce the risk of CMs overall, or the risk of a specific and selected group of them [38], namely oral clefts [39-42], cardiac defects [43-46], urinary tract anomalies except hypospadias [47], limb reduction defects [48], omphalocele [49], anal atresia [50].

Because more than 10,000 children are estimated to be born daily with CMs worldwide, even a 15-20% decrease in the overall risk for birth defects would be important.

The preventive effect of the FA might therefore affect several CMs, as suggested by the Hungarian cohort-controlled trial [35], and a case–control study conducted in Atlanta, United States [36]. These studies observed a reduction of 47% and 16% respectively in all CMs, except NTDs. We underline that the Hungarian study was conducted with 0.8 mg/day and the American one with an average dose of 0.4 mg/day. It is reasonable to hypothesize that the percentage of CMs preventable with a dose of 0.4 mg/day is 20%, and with 4 mg this is at least 20% greater.

The Hungarian study conducted also shows a 60% risk reduction of the Down syndrome (OR = 0.4, 95% CI 0.2-0.7) in women who had taken 6–9 mg/day of FA before pregnancy [51]. This association was not confirmed in other conditions [52,53].

Unfortunately, from the observational studies it is impossible to gain a precise estimate of the reduction associated with the FA supplementation. A robust study design as is obtained in an RCT is needed to confirm these results and obtain a more precise size effect.

Furthermore, the effects of FA supplementation for the prevention of other infant and maternal outcomes are unclear.

In 2005 a large RCT of FA supplementation found no evidence of an effect of supplements on mean birth weight, placental weight or gestational age at delivery. There was a possible reduction in risk of low birth weight or antepartum haemorrhage with high-dose FA supplements, and a possible reduction in risk of pre-eclampsia with low and high dose [54].

In the recent Cochrane meta-analysis of randomized and quasi-randomized trials, there were no significant differences between experimental and control groups on other pregnancy outcomes [33]. Considering the supplementation with FA versus any other interventions/placebo, all five trials examined the rate of miscarriage in women with confirmed pregnancy. While the number of miscarriages increased in the group receiving supplements containing FA, the difference between groups did not reach statistical significance (RR = 1.10, 95% CI 0.97-1.26). The number of babies with low birth weight (less than 2500 g) was examined in one trial (186 babies). There was no significant evidence of a difference between treatment groups. Stillbirths were reported in four trials. There was no significant evidence of any difference between treatment groups in the pooled analysis (RR = 0.96, 95% CI 0.51-1.83). The number of women affected by multiple pregnancy (2 or more foetuses at birth) was examined in three trials. Overall there was no evidence of any significant difference between women receiving supplements with FA and those in the controls (RR = 1.32, 95% CI 0.88-1.98). No trials reported results for preterm birth (less than 37 weeks’ gestation) and pre-eclampsia, defined as gestational hypertension (blood pressure higher than 140/90 mmHg) and proteinuria (more than 300 mg of protein in a 24-hour urine sample). All of the studies included were published before 2001.

Observational studies have suggested that a higher level of folate in pregnancy is associated with higher birth weight, increased placental weight and fewer preterm births [28,55-57], although these results have not been confirmed in other populations recently [58]. At present the association between folate insufficiency and the onset of placenta-mediated diseases, such as spontaneous abortion [58-65], preterm birth [66-75], foetal growth retardation [76-80], pre-eclampsia [81-88], abruptio placentae [89] and stillbirth [90] is still not entirely clear.

Moreover, prenatal multivitamins containing FA appear to be associated with a significant protective effect on three paediatric cancers: leukaemia (OR = 0.61, 95% CI 0.50-0.74), paediatric brain tumours (OR = 0.73, 95% CI 0.60-0.88) and neuroblastoma (OR = 0.53, 95% CI 0.42-0.68) [91,92]. Recently, ecological studies provided support for a decrease in Wilms tumour, neuroblastoma, primitive neuroectodermal tumours and ependymomas after Canadian and United States FA fortification [93-95].

It is also of interest to investigate whether maternal FA supplementation during pregnancy may be associated with a reduced risk of other neurodevelopmental disorders in children [96,97]. A recent study of 38,954 children in the Norwegian Mother and Child Cohort Study found that peri-conceptional maternal intake of FA supplements was associated with a lower risk of severe language delay at age 3 years [98]. In the same cohort, the use of FA supplements around the time of conception was associated with a lower risk of autistic disorder [99].

#### Peri-conceptional FA supplementation

Supplementation with FA before the time of conception reduces the risk of NTDs. This protective effect has led to mandatory flour fortification with FA in several countries [100], and it is generally recommended that women planning to become pregnant take a daily supplement of FA.

These findings led the Public Health Authorities in some countries (e.g.: United States [101], Canada [102], Chile [103], Costa Rica [104], South Africa [105], Australia [106]) to fortify staple food [107-109] and the Departments of Health of many other countries (mainly in Europe [110,111]) to recommend that women of childbearing age consume an extra 0.4 mg of FA per day to prevent NTDs.

Supplementation with FA is internationally recommended to women from the moment they try to conceive until 8–12 weeks of pregnancy. At present the most common public health recommendation to prevent the occurrence of NTDs and possibly other CMs is to take an FA supplement containing 0.4 mg/day [111]. However, the debates about the most appropriate dose and increasing FA supplementation in childbearing age are still unresolved. With respect to FA dosage, a review of efficacious doses conducted by Wald et al. [112] suggested a dose-dependent effect.

It must be realized that the two RCTs clearly showing a reduced risk of NTDs were performed with 4.0 mg/day [29] and 0.8 mg/day [30] of FA. Furthermore, a non-randomized study on the recurrence risk of oral clefts observed a risk reduction of 66% using a FA peri-conceptional supplementation of 10 mg/day [113]. A Hungarian study showed that only a high dose (6 mg/day) of FA reduced the risk of oral clefts by 25% (OR = 0.75, 95% CI 0.58-0.96) but not a lower dose (<1 mg/day) [114], and a Hungarian case–control study on Down syndrome observed a decreased risk of 60% (OR = 0.4, 95% CI 0.2-0.7) after the pre-conceptional use of a high dose of FA (6–9 mg/day) [51].

In 1995 Daly et al. showed that NTDs risk was associated with red blood cell (RBC) folate levels in a continuous dose–response relationship [115]. In this case–control study, the group of women with the lowest NTDs risk equal to 0.8 per 1,000 births was the group with RBC folate levels equal to or higher than 906 nmol/L, with a group mean of 1,292 nmol/L. According to other experts [116,117], the concentration of RBC folate of 906 nmol/L can be considered the most desirable level in early pregnancy to reduce the NTDs risk.

However, studies conducted in Europe failed to find an optimal level of serum folate in these populations [112,118] and recent data suggest that pregnant women use high doses of FA supplementation in early- to mid-pregnancy in several European countries [75,79,119].

The standard recommendation for the recurrence prevention of NTDs and to prevent their occurrence in epileptic and diabetic mothers is to take a higher dose of FA, 4–5 mg/day [111]. Furthermore, in 2007 the Canadian Recommendations also included obesity (BMI >35) as a health risk and recommended "the higher dose FA strategy (5 mg)" in patients with a history of poor compliance with medications and additional lifestyle issues of variable diet, no consistent birth control, and alcohol, tobacco, and recreational non-prescription drugs use [120,121]. In particular, the use of FA supplements might (partially) neutralize the adverse effects of smoking on DNA methylation, since folate provides methyl groups for the synthesis of methionine and plays a critical role in homocysteine metabolism [122-124].

Moreover, women planning to become pregnant might be exposed to medications with known antifolate activities affecting different parts of the FA metabolic cascade. Epidemiological studies have shown an increased risk of birth defects or placenta-mediated adverse pregnancy outcomes among women exposed in early gestation to antiepileptic drugs (carbamazepine, valproate, barbiturates), sulphonamides or methotrexate [125,126]. Hence, whenever women use these medications, or have used them in the period prior to conception, they should take 5 mg/day of FA until the end of the first trimester of pregnancy [121].

Wald et al. [112] addressed the issue of the efficacy of high FA dosage more comprehensively, assessing the effect of increasing FA intake on serum folate levels and on the relation between these levels and the risk of having NTDs during pregnancy. They found that from a typical western background serum folate level of 5 ng/mL, an increase of 0.4 mg/day would reduce the risk of NTDs by about 36%, while taking 5 mg/day would reduce the risk by about 85%. A working group convened by the WHO Regional Office in Europe and Istituto Superiore di Sanità on 11–12 November 2002 endorsed these estimations [127].

Actually there is no good evidence to advise a different dose to women without an increased risk, women with a foetal NTD during a previous pregnancy or women with another well-known risk factor. If a dose of 4–5 mg/day can prevent recurrence or occurrence in women with well-known risk factors, it should be the same for the occurrence and in women with no known risk factors.

The question remains concerning the best dose of FA supplementation, as the prevalence of CMs worldwide is still high.

Overall, from all these studies it can be speculated that an increased peri-conceptional intake of FA can cause a greater reduction in the risk of NTDs as well as a greater and measurable reduction in the risk of other CMs, including trisomy 21 syndrome.

The hypothesis that a higher intake of FA is related to a higher risk reduction of NTDs and of other CMs is the main rationale for the Italian and Dutch RCTs.

#### FA supplementation in second and third trimester of pregnancy

During pregnancy, increased folate intake is required for rapid cell proliferation and tissue growth of the uterus and placenta, growth of the foetus, and expansion of the maternal blood volume.

Continued FA supplementation with 0.4 mg/day in trimesters 2 and 3 of pregnancy can increase maternal and cord blood folate status and prevent the increase in homocysteine concentration that otherwise occurs in late pregnancy [128].

However, the effect of folate supplementation throughout pregnancy on several other health outcomes is unclear. Observational studies suggest a potential benefit of good maternal folate status on birth weight, placental weight or length of gestation [129,130]. In contrast, supplementation trials have shown equivocal results.

The meta-analysis of Fekete et al. [131] demonstrated significant dose–response relationship between folate intake and birth weight. However, the results indicated no evidence of any effect of supplementation on placental weight or length of gestation.

In 2013 the Cochrane meta-analysis [132] conducted to assess the effectiveness of FA supplementation during pregnancy on maternal health and pregnancy outcomes provided non-conclusive evidence of the benefits of supplementation on pregnancy outcomes: there was no impact on preterm birth (RR = 1.01, 95% CI 0.73-1.38) or stillbirths/neonatal deaths (RR = 1.33, 95% CI 0.96-1.85). However, improvements were seen in the mean birth weight (mean difference (MD) = 135.75, 95% CI 47.85-223.68). The included studies did not report on the impact of FA supplementation on miscarriage or pre-eclampsia.

Pre-eclampsia is a leading cause of indicated preterm delivery and accounts for 25% of very low birth weight infants. Furthermore, pre-eclampsia may also increase the risk of cardiovascular disease in the offspring through "foetal origin of adult diseases" [133] and women with a history of pre-eclampsia continue to be at increased risk for future cardiovascular events [14,15]. As the review of Wen et al. [134] suggests, while earlier studies showed protective effect of FA on reduced risk of pre-eclampsia [81-84], some recent studies failed to find such an effect [87,88]. Selection bias, confounding factors and the low dose of FA used could account for these differences. Given the disease burden of pre-eclampsia, novel preventions such as FA need to undergo proper scientific investigation [134].

The relative paucity of data indicates that there is an urgent need to develop further studies focusing on health outcomes of folate supplementation after the first trimester. More well-designed, large-scale RCTs are needed to establish the benefits of FA during pregnancy before supplementation throughout pregnancy can be recommended on these grounds.

The Dutch and Italian trials might provide useful information to clarify this possible association.

### Safety issue

The safety of FA supplementation has been the subject of some debate. When focusing on side effects in the women of childbearing age, the target population of pre-conceptional supplementation programmes, the only side effect to be considered is an increase of dizygotic twins, as suggested by a Swedish study [135] which, however, did not sufficiently allow for in vitro fertilization (IVF) and sub-fertility treatments [136]. Moreover, a Norwegian study, analogous to the Swedish one, showed that the association between peri-conceptional FA supplementation and dizygotic twins disappeared after exclusion of known IVF pregnancies, and accounting for underreporting of both IVF pregnancies and folate use [137]. The meta-analysis of Muggli et al. [138] suggests that the hypothesis of the increase in dizygotic twins is still to be demonstrated (OR = 1.26, 95% CI 0.91-1.73 for pre-conceptional supplementation and dizygotic twins; OR = 1.02, 95% CI 0.85-1.24 for overall twins), that it would need more data to be seriously considered and, if true, it would only cause a very limited increase.

Moreover, questions have emerged about the role of in utero exposure to folate through maternal intake and the development of asthma and atopic disease [139]. Causes of asthma are considered to be an interaction of both genetic and environmental risk factors, and the impact of nutrition and other exposures during pregnancy on long-term health and development of children has been of increasing interest. The meta-analysis from the National Center on Birth Defects and Developmental Disabilities in Atlanta, United States [140] provided no evidence of an association between maternal FA supplement use (compared with no use) in the pre-pregnancy period through the first trimester and asthma in childhood (summary risk estimate = 1.01, 95% CI 0.78-1.30), although this finding was limited by the small number of studies.

In general, population concerns about FA were mostly related to the effect of masking pernicious anaemia due to B12 deficiency and to the possible effects on cancer. Current exposure to FA through fortification in the United States has been reported not to increase the risk of masking anaemia [141]. Regarding the cancer, to see whether the randomized trials of FA show an increase or decrease in cancer risk over a period of just a few years, Vollset et al. [142] conducted collaborative meta-analyses of site-specific cancer incidence during the scheduled treatment period among 50,000 individuals from all available large cardiovascular and adenoma trials. They found that FA supplementation does not substantially increase or decrease incidence of site-specific cancer during the first 5 years of treatment (RR = 1.06, 95% CI 0.99-1.13).

## Objectives

This project is a joint effort by research groups in Italy and the Netherlands. In both countries, the additional effect of peri-conceptional FA supplementation of 4 mg/day compared to the 0.4 mg/day standard dose will be assessed. In the Netherlands, the effect of continued FA supplementation in the second and third trimesters of pregnancy will be studied.

### Italian RCT

The primary objectives are:

• to promote the implementation of pre-conceptional counselling in a formal and structured way and with defined procedures, to support a wider prevention of CMs and maternal-infant health;

• to assess the effect of peri-conceptional FA supplementation of 4 mg/day compared to the 0.4 mg/day standard dose on reducing the occurrence of CMs.

Other aims of this study include comparing severity of CMs in offspring of trial mothers, rates of selected CMs, rates of miscarriage, pre-eclampsia, preterm birth, small for gestational age, abruptio placentae.

### Dutch RCT

The primary objectives are:

• to assess the effect of peri-conceptional FA supplementation of 4 mg/day compared to the 0.4 mg/day standard dose on the prevalence of CMs;

• to assess the effect of 0.8 mg *vs* 0.2 mg FA supplementation in the second and third trimesters of pregnancy on the prevalence of preterm birth and pre-eclampsia.

The project is currently going on. Despite a specific recruitment strategy in both countries, the lack of acknowledged clinical activity defined as preconceptional counselling, together with the absence of a routine setting which women planning pregnancy naturally refer to, the study cannot maintain the planned trend of recruitment.

Nonetheless, considering the relative paucity of data and the increasing interest toward the effect of FA also on other end points of the pregnancy, the need to develop further studies focusing on health outcomes of folate supplementation, together with difficulties in subjects recruitment, the research group is still engaged in the assessment of the effect of peri-conceptional FA supplementation of 4 mg/day compared to the 0.4 mg/day standard dose in reducing the occurrence of the main adverse pregnancy outcomes. The following were grouped together: CMs, miscarriage, pre-eclampsia, preterm birth, small for gestational age, abruptio placentae.

## Methods/Design

### Study design

#### Italian RCT

The present study is a randomized, double-blind, controlled trial evaluating whether supplementation with 4 mg/day of FA reduces the overall risk of major adverse pregnancy outcomes in the population in comparison to the standard recommended dose of 0.4 mg/day.

#### Dutch RCT

The study is an RCT, with four intervention arms. Initially, two groups are formed: 4.0 mg FA *vs* 0.4 mg FA supplementation peri-conceptionally and both of those groups will be split at 12 weeks of gestation into a group with 0.2 mg FA until the end of pregnancy and a group with 0.8 mg FA until the end of pregnancy:

1) 0.4 mg of FA from 4 weeks pre-conception to 12 weeks of gestation, and 0.2 mg FA supplementation after 12 weeks of gestation;

2) 0.4 mg of FA from 4 weeks pre-conception to 12 weeks of gestation, and 0.8 mg FA supplementation after 12 weeks of gestation;

3) 4.0 mg of FA from 4 weeks pre-conception to 12 weeks of gestation, and 0.2 mg FA supplementation after 12 weeks of gestation;

4) 4.0 mg of FA from 4 weeks pre-conception to 12 weeks of gestation, and 0.8 mg FA supplementation after 12 weeks of gestation.

### Study population

#### Italian RCT

Women of childbearing age, 18–44 years, who intend to become pregnant within 12 months are eligible for the study. All eligible women who show up at obstetric and gynaecological clinics, assisted reproduction centres, family planning services, general practitioners and paediatricians are invited to visit the randomization centres for a complete pre-conception counselling and a talk about participating in the study.

Women are excluded if they: (a) are pregnant, (b) do not intend to become pregnant, (c) are younger than 18 and older than 44 years, (d) are planning to move to an area where the study is not implemented, (e) do not understand Italian, (f) do not have a phone, (g) are affected by epilepsy, even not treated with anti-convulsant drugs, (h) are affected by diabetes, (i) have suffered from cancer or a serious disease (e.g.: Crohn disease, rheumatoid arthritis, ulcerative colitis), (j) are being treated with antifolates or have recently been treated with it, like methotrexate, (k) abuse or have abused alcohol, (l) are obese (BMI ≥30), (m) are vegetarian, (n) had a previous pregnancy with NTD or any other congenital structural birth defects, (o) are affected or have a partner that is affected by NTD, have a relative (in her family or in the partner’s family) affected by NTD, (p) have a family medical history of breast cancer or colorectal cancer, (q) have a personal or family history of an hereditary syndrome like familiar adenomatous polyposis or hereditary nonpolyposis colorectal cancer, (r) report allergy to FA, (s) present contraindications to FA use, (t) are affected by megaloblastic anaemia, (u) take defined dosages of FA for directions other than those listed in the above exclusion criteria.

#### Dutch RCT

All women living in the Northern region of the Netherlands aged between 18 and 45 years who want to become pregnant within 12 months are eligible for participation in the study.

Women are excluded if they: (a) had previous offspring with NTD, (b) do not give informed consent, (c) do not understand Dutch, (d) plan to move to an area where the study is not implemented, (e) use FA antagonists, (f) are affected by diabetes, megaloblastic anaemia and/or cancer (previous cancer or abnormal PAP smears), (g) are already pregnant at the time of inclusion or within 4 weeks after the start of the intervention, (h) take high doses of FA for any other reason.

### Recruitment

#### Italian RCT

In Italy, as well as in other countries, there is no recognized clinical activity defined as "pre-conceptional counselling". All women planning their pregnancy who discuss their intention to hospital and community gynaecologists, general practitioners, paediatricians, and obstetricians in the various areas of Italy are invited to refer to the randomization centres to receive fully structured pre-conceptional counselling according to the standard recommendations and, if consenting, to be recruited into the study and randomized.

The invitation is particularly focused on making the woman and the couple aware of the importance of complete pre-conceptional counselling, as a means of reducing possible adverse events in the human reproduction process. FA supplementation is one of the actions promoted by the pre-conceptional counselling. The recruiting investigators explain such preventive intervention and its objectives to the women and highlight the possibility to participate to the randomized study. The invitation to contact the randomization centres is reinforced by an ad hoc informative sheet.

The randomization centres are obstetric and gynaecological clinics, assisted reproduction centres, family planning services, and general practitioners.

#### Dutch RCT

In the Netherlands, eligible women are primarily approached through proactive recruitment campaigns conducted by community pharmacies (CPs). Inclusion takes place in a coordinated campaign run by the community pharmacists and the general practitioner of the participant.

Since individual pre-conception care is currently not available on a large scale in the Netherlands, no "natural" existing starting point for the recruitment procedure is available. The target population from which the actual recruitment has to take place consists of all women of childbearing age that have the intention to become pregnant within the next 12 months. The actual inclusion (final step of the recruitment process) mainly takes place during CP visits, in which consent is given.

Personal informative mailings from CPs are sent to all female clients in the age group 25–35, accompanied by a letter that requests, if appropriate, considering participation in the trial on behalf of the pharmacist. In addition, permanent public information are present in the CP and recurrent personal information are given at delivery of certain Over-The-Counter and prescribed drugs in the CP. Other health professionals contribute towards informing the target group: posters and leaflets are available at well baby clinics and with primary care physicians. At some sport schools and child care centres posters are present. Representatives of the research team promoted participation in the trial and gave information at fairs targeted at women of reproductive age.

### Randomization procedures

#### Italian RCT

After giving informed consent, women are randomly assigned to receive a pill containing 4 mg of FA (treatment in study) or 0.4 mg of FA (referent treatment) daily. The randomization code is generated by a web-based patient randomization system, stratified according to the enrolment site and the maternal age group. The study is double-blinded: subjects, investigators and research staff are blinded to the randomization assignments.

#### Dutch RCT

After giving informed consent, women are randomized to one of the four groups. Randomization is not stratified. Every four participants per research centre are assigned to a different study group, resulting in equal distributions among the study groups. The study is double-blinded: subjects, investigators and research staff are blinded to the randomization assignments.

### Interventions

#### Italian RCT

At the enrolment visit, full pre-conceptional counselling is offered to women according to standard guidelines. All the eligible women are interviewed by the investigators using a structured form to evaluate socio-demographic and lifestyle characteristics, past and present health status, previous pregnancy outcomes, and the previous four months of food intake.

Subjects who show interest in participating in the study and who meet the inclusion criteria, in the absence of exclusion criteria and after giving informed consent, are randomized to either 4 mg or 0.4 mg of FA.

All women are given a box containing 130 pills, according to the blinded randomization arm. All women are then recommended to take one pill per day and to contact the enrolment centre when only 20 pills are left in the box so that they get another box of 130 pills. After randomization, enrolled women are interviewed every 4 months by the investigators (when they contact the randomization centre to get another box of FA pills), to evaluate the pregnancy status.

If women become pregnant, they will take the FA until the end of the 12th completed gestational week. If they have not become pregnant after 12 months of FA supplementation, the women will be removed from the study.

#### Dutch RCT

Women in all intervention groups receive identical pills, containing two different doses of FA (4 or 0.4 mg). Women start taking the pills after randomization, but at least 4 weeks before conception, and will receive new pills from their pharmacy every 4 months.

At 12 weeks of gestation, all women will receive a new set of pills, half of them receiving 0.2 mg supplements and half 0.8 mg of FA. Again the pills are identical. New pills are issued once more during pregnancy (25 weeks of gestation).

### Information collected and follow-up

#### Italian RCT

At the enrolment visit, all the eligible women are interviewed by the investigators using a structured form to evaluate socio-demographic and lifestyle characteristics, past and present health status, previous pregnancy outcomes, and the previous four months of food intake.

After randomization, enrolled women are interviewed by the investigators every 4 months (if not pregnant), when they contact the randomization centre to get another box of FA pills. The interview at 4-8-12 months is performed using a structured form to evaluate the pregnancy status, current health conditions, and experimental medicine intake.

Enrolled randomized women who are diagnosed as being pregnant during the study period and who take the FA until the end of the 12th completed gestational week, are invited to come back to the randomization centre to return the remaining pills and to update their status.

They will be interviewed at the expected 16, 24 and 40 weeks of gestation by a trained Health Care Provider (HCP) by phone using a structured form to evaluate the pregnancy outcome. All the pregnancy outcomes, irrespective of the information reported by the woman at the interview, will be checked in the hospital’s clinical records by a trained HCP.

The health status of live births will be evaluated by a trained HCP through an interview with the attending physician using a structured questionnaire when the child reaches the age of 1 month, 3 months and 1 year, and an interview with the parents when the child is 1 year old.

#### Dutch RCT

Most data are gathered through questionnaires in the Netherlands. Information on all CMs of live births, stillbirths and terminations of pregnancy following prenatal diagnosis are derived from the database of EUROCAT, where virtually all CMs are registered. Data about the diagnosis and the medical history are collected in a standardized procedure of high quality.

Variables derived from medical records are: birth weight, gestational age, diseases during pregnancy (diabetes, high blood pressure), abruptio placentae, Apgar score.

In a short questionnaire, women answer questions on compliance to trial medication, the use of vitamin supplements and the use of alcohol, tobacco and drugs. These questions will be repeated each time they pick up their supplements at the pharmacy.

Follow-up: after randomization, women receive a new set of pills every 4 months at the pharmacy, until a period of 12 months has elapsed without them getting pregnant or until the end of their pregnancy (live birth, stillbirth, spontaneous abortion or termination). After the pregnancy has been completed, women and their general practitioners will receive a final questionnaire about pregnancy outcomes by mail.

### Definition of treatment compliance and withdrawal

#### Italian RCT

Compliance to FA supplementation is evaluated by counting the pills not taken and that remain in the given box at the randomization centre.

Three categories will be defined for compliance status: (a) fully treated women: those women who will take at least 70% of the expected pills during the full peri-conceptional period (1 month before and 3 months after last menstruation); (b) partially treated women: women who will take between 30% and 69% of the pills during the full peri-conceptional period, (c) non-treated women: less than 30% of the pills. The last category will be considered as non-compliant.

Patients may be withdrawn and excluded from the "protocol analysis" but evaluated at the "intention-to-treat" analysis for any of the following reasons: any protocol violation, non-compliance, lost to follow-up, patient withdrawn at her own request, for reasons other than those above.

#### Dutch RCT

Women are asked to return all pills they have not used to the pharmacy every time they collect a new set of pills, in order to check compliance with FA supplementation.

All analyses will be performed according to the intention-to-treat principle. In addition, alternative per protocol analyses will be performed, excluding all participants who were not compliant with FA supplementation. For the analysis on the effect of FA supplementation on CMs, groups 1 and 2 (low dose) and 3 and 4 (high dose) will be combined, since no interaction between early FA and late FA supplementation is expected.

### Outcome measures

#### Italian RCT

The primary study outcome is the total number of adverse pregnancy outcomes as listed above.

#### Dutch RCT

The primary outcomes include all FA-related CMs.

Preterm birth is defined as a gestational age <37 weeks.

Pre-eclampsia is defined as a systolic blood pressure ≥ 140 mmHg and/or diastolic blood pressure ≥ 90 mmHg after 20 weeks of gestation among women with previously normal blood pressure, combined with proteinuria (≥300 mg/24 hours).

### Sample size

In the original protocols, sample size was computed considering CMs as end point. However, given the increasing interest toward the effect of FA supplementation on all pregnancy outcomes on the one hand, and the difficulties encountered in subjects recruitment on the other hand, we foresee to analyze the data considering all the adverse outcomes of pregnancy taken together in a global end point (e.g.: CMs, miscarriage, pre-eclampsia, preterm birth, small for gestational age, abruptio placentae).

Since the frequency of the cumulative group of adverse pregnancy outcomes has an estimated frequency of 30% (unpublished observation), a total of about 1,000 pregnancies need to be evaluated to detect an absolute reduction of the frequency of 8% with allocation ratio 1:1, alpha error of 0.05, power of 80%.

After one year of pregnancy planning or active pregnancy search, a pregnancy rate of 80-85% is expected. Since the sample size needed to analyze outcomes separately is large, this project also promotes an international prospective meta-analysis.

### Data analysis

The analysis will be conducted by protocol and by intention to treat. A multivariate model will be used where unbalanced characteristics will be considered. A subgroup analysis will be performed for women with assisted reproduction.

### Ethical considerations

#### Italian RCT

The project has been approved by the ethical committee of the randomization centres, and all subjects provide written informed consent.

#### Dutch RCT

The project has been approved by the ethical committee of the VU University Medical Center, and all subjects provide written informed consent.

### Data Safety Monitoring Committee

#### Italian and Dutch RCT

The study will be interrupted if:

• there is evidence that the study drug causes suspected unexpected serious adverse reactions (SUSAR) or serious adverse events (SAE) in the short or long term;

• the frequency of any reproductive adverse event or pregnancy complication in the overall group of women in the study is higher than the expected frequency.

To regularly check this, the Data Safety Monitoring Board (DSMB) will evaluate the global frequency of reproductive adverse events or pregnancy complications in the total sample of randomized women, and will communicate it to the General Coordination Centre (GCC).

### Relevance for society

Between 200,000 and 300,000 cases of NTDs are born each year worldwide. Other CMs that might be prevented with FA supplementation are heart anomalies, limb defects, urinary tract malformations, oral clefts and Down syndrome. These anomalies far outnumber the figures for NTDs. It must be stressed that the potential impact on child health is huge. Even a 13% reduction of all CMs means 39,000 healthy infants per year out of 10 million births in the WHO European Region. The most common and severe CMs are included among a selected group that may be more sensitive to the preventive action of FA (NTDs, oral clefts, cardiac defects, urinary tract anomalies, limb reduction defects, omphalocele, anal atresia). Even an apparently small decrease (e.g.: 20-30%) of the frequency of all CMs through a primary prevention intervention is an extremely important public health issue, since we can secure the health of a great number of children that would otherwise be affected as well as save money.

All these CMs have a huge impact on the health, quality of life, and life expectancy of the children. They also have a major impact on the lives and quality of life of their family. If the preventive effect of FA for CMs other than NTDs were to be proven, this fact could be used to further conduce the pre-conceptional use of FA supplements through patient education strategies, thereby increasing the overall effect of FA prevention. If higher doses of FA supplementation appear to be more effective than the now recommended daily dose of 0.4 mg, this would be an improvement of an existing intervention with huge impact.

But it is not only CMs that might be affected by FA supplementation. The risk of pre-eclampsia and preterm birth is also likely to be reduced by FA supplementation. The prevention of both CMs and preterm birth will result in a reduced perinatal death rate.

The proof of additional preventive effects of FA over the currently accepted effects of 0.4 mg on NTDs only has a significant impact on the need for, and the possibilities to reduce, social inequalities in health.

## Discussion

In conclusion, the total group of adverse pregnancy outcomes is the single major cause of infant morbidity, mortality and disability in all countries. Therefore even an apparently small decrease (e.g.: 20-30%) of the frequency of major adverse pregnancy outcomes through a primary prevention intervention is an extremely important public health issue, since we can secure the health of a great number of children who would otherwise affected. Prevention in this field is therefore highly important.

We designed an RCT to assess the effect of FA peri-conceptional supplementation of 4 mg/day compared to the 0.4 mg/day standard dose on reducing the occurrence of major adverse pregnancy outcomes. The project will also give the opportunity to promote fully standardized pre-conceptional counselling in all the women contacted to participate and then to promote a wider prevention of adverse pregnancy outcomes and maternal-infant health.

## Abbreviations

BMI: Body mass index; CI: Confidence interval; CMs: Congenital malformations; CP: Community pharmacy; DSMB: Data safety monitoring board; EUROCAT: European surveillance of congenital anomalies; FA: Folic acid; GCC: General Coordination Centre; HCP: Health care provider; IVF: In vitro fertilisation; MD: Mean difference; MRC: Medical Research Council; NTDs: Neural tube defects; OR: Odds ratio; RBC: Red blood cell; RCT: Randomized clinical trial; RR: Relative risk; SAE: Serious adverse event; SUSAR: Suspected unexpected serious adverse reaction.

## Competing interests

The authors declare that they have no competing interests.

## Authors’ contributions

*Italian RCT:* RB, FF, EL, PM are all members of the General Coordination Centre of the Italian folic acid trial study group. PM conceived the study. RB and PM participated in the project design, wrote the protocol and wrote the current manuscript. RB and FF coordinate the development of the study. PM and EL are in charge of the statistical analysis. PB, FF and EL critically reviewed and revised the manuscript. *Dutch RCT:* FB, MvP, DS, MC, and HdW are all members of the General Coordination Centre of the FoliumzuurExtra study group. MC, DS, and MvP conceived the Dutch study and participated in the project design. DS coordinated the execution of the recruitment strategy and lead the logistics of the intervention implementation. FB executed the study. All authors have read and approved the final version of the manuscript.

## Authors’ information

Italian RCT

Office for Research Promotion, Department of the Hospital Management and Pharmacy, Verona University Hospital, Verona; Alessandra Lisi International Centre on Birth Defects and Prematurity-ICBD, WHO Collaborating Centre, Rome; Healthcare Directorate, Verona University Hospital, Verona.

Dutch RCT

Community Genetics, Department of Clinical Genetics, EMGO Institute for Health and Care Research, VU University Medical Center, Amsterdam; Department of Public and Occupational Health, EMGO Institute for Health and Care Research, VU University Medical Center, Amsterdam; MediClara Projects BV, Abcoude; Department of Genetics, University Medical Center Groningen, University of Groningen, Groningen, the Netherlands.

THE ITALIAN FOLIC ACID TRIAL STUDY GROUP:

General Coordination Centre: Renata Bortolus, Francesca Filippini, Alessandra Compagni, Erika Rigotti, Antonella Noya di Lannoy (Verona University Hospital, Verona), Pierpaolo Mastroiacovo, Emanuele Leoncini (ICBD, Rome).

Experimental Medicine Management: Alessandra Compagni, Marilisa Coati, Silvia Manfrè, Roberto Barbazza (Verona University Hospital, Verona).

Study Investigators and Randomization Centres: Giovanni Zanconato, Maria Teresa Zenorini, Vittorio Travagliati, Elena Mantovani, Anna Angeli, Elena Cavaliere, Greta Cherubini, Alice Negretto, Elena Lavarini, Milena Ozzi, Nikolaos Papadopoulos (Verona University Hospital), Enrico Di Mambro, Maddalena Vessella (Adria Hospital), Giuseppe Ettore, Sebastiano Bianca, Chiara Barone (ARNAS Garibaldi Nesima, Catania), Erich Cosmi, Silvia Visentin, Martina Camerin (Azienda Ospedaliera of Padua), Paola Lanza (Bassano del Grappa Hospital), Simonetta Marinangeli (Family Planning Service, Bassano del Grappa), Giorgia Negrini, Alberto Ottaviani, Laura Zivelonghi (Bussolengo Hospital), Andrea Baffoni, Michaela Bertezzolo, Mara Pistolato (Conegliano Hospital), Enrico Ioverno (General Practitioner, Dueville), Edgardo Somigliana, Claudia Scarduelli, Federica Alagna, Giulia Santi (Fondazione IRCCS Cà Granda Ospedale Maggiore Policlinico, Milan), Elena Cesari (Gallarate Hospital), Paola Zanini, Achille Morandini (Legnago Hospital), Irene Cetin, Arianna Laoreti (Luigi Sacco Hospital, Milan), Chiara Tresso (General Practitioner, Malo), Maria Grazia Salviato (Family Planning Service, Martellago), Marina Matterazzo (Family Planning Service, Mira), Carlo Failli (Family Planning Service, Montebelluna), Maurizia Marzolini (Family Planning Service, Oderzo), Anna Casaro (Family Planning Service, Padua), Debora Balestreri, Elena Benassi, Elisa Caloi (S. Bonifacio Hospital), Francesco Libero Giorgino, Alessandra Schiavo (Family Planning Service, San Pietro di Stra), Gian Pietro Piazza (General Practitioner, Schio), Renato Ruffini (General Practitioner, Sovizzo), Gianfranco Jorizzo, Gaetana Cirelli, Francesca Arcidiacono, Anna De Toni, Silvia Rusconi (Thiene Hospital), Claudia Guaraldi (Valdagno Hospital), Patrizia Rosi, Graziella Mortaro (Family Planning Services, Verona), Laura Valotto, Angelo Guido (General Practitioners, Verona), Giuliano Zanni, Chiara Vernier (Vicenza Hospital), Anna Sandri (Family Planning Service, Vigonovo), Nedelia Minisci (Family Planning Service, Villorba).

THE DUTCH FOLIC ACID TRIAL STUDY GROUP:

General Coordination Centre: Martina Cornel, Mireille van Poppel, Fenneke Blom, Tamar Kruit (VU Medical Center, Amsterdam), Denhard de Smit (MediClara Projects BV) Hermien de Walle (UMCG, Groningen).

Research Centres (Community Pharmacies): Apotheek Bonnehûs, Leeuwarden; Apotheek Boterdiep, Groningen; Apotheek de Wijert, Groningen; Apotheek Delfzijl, Delfzijl; Apotheek Diephuis, Groningen; Apotheek Eemsmond, Uithuizen; Apotheek Greving, Leeuwarden; Apotheek Hoving, Bergum; Apotheek It Krúswâld, Kollum; Apotheek It Krúswâld Buitenpost; Apotheek Marum, Marum; Apotheek Neptunus, Delfzijl; Apotheek Salentijn, Appingedam; Apotheek Sappemeer, Sappemeer; Apotheek Scheemda, Scheemda; Apotheek Schoterpoort, Heerenveen; Apotheek Stadskanaal, Stadskanaal; Apotheek ‘t Hooge Zand; Apotheek Wildersgang, Delfzijl; Apotheek Winsum, Winsum; BENU Apotheek A-straat, Groningen; BENU Apotheek Borger, Borger; BENU Apotheek de Peeleres, Assen; BENU Apotheek Gorredijk, Gorredijk; BENU Apotheek Klazienaveen, Klazienaveen; BENU Apotheek Vredeveld, Assen; BENU Apotheek Workum, Workum; BENU Apotheek Zultherveld, Roden; Boots Apotheek Lewenborg, Groningen; Gezondheidscentrum Camminghaburen, Leeuwarden; Kring-apotheek Beijum, Groningen; Kring-Apotheek de Dorpsacker; Kring-apotheek Dokkumer Wâlden; Kring-apotheek Haskerbrug, Heerenveen; Kring-apotheek Karsten, Assen; Kring-Apotheek Postma, Sneek; Kring-apotheek Selwerd, Groningen; Kring-apotheek Stiens, Stiens; Kring-apotheek Swarte, Heerenveen; Kring-apotheek Zuidlaren, Zuidlaren; Linde Apotheek, Heerenveen; Mediq Apotheek Barentsen, Drachten; Mediq Apotheek Bedum, Bedum; Mediq Apotheek de Hondsrug, Emmen; Mediq Apotheek De Rietlanden, Emmen; Mediq Apotheek Haren, Haren; Mediq Apotheek Harkema, Harkema; Mediq-Apotheek Surhuisterveen, Surhuisterveen; Medsen Apotheek Hardegarijp, Hardegarijp; Peizer Apotheek, Peize; Pekelder Service-Apotheek, Nieuwe Pekela; Pekelder Service-Apotheek, Oude Pekela; Service-Apotheek Bilgaard, Leeuwarden; Service-Apotheek Boiten, Leeuwarden; Service-Apotheek De Griffioen, Wolvega; Service-Apotheek de Wissel, Noordwolde; Service-Apotheek Dokkum bij het Ziekenhuis, Dokkum; Service-Apotheek Dokkum Centrum, Dokkum; Service-Apotheek Eelde-Paterswolde, Paterswolde; Service Apotheek Emmen, Emmen; Service Apotheek Grootegast, Grootegast; Service-Apotheek Hanzeplein, Groningen; Service-Apotheek Hoogkerk, Groningen; Service-Apotheek Kranenborg, Hoogezand; Service-Apotheek Musselkanaal, Musselkanaal; Service-Apotheek Sasburg, Sneek; Service-Apotheek van der Sluis, Sneek; Service-Apotheek Themmen, Assen; Service-Apotheek West BV, Wolvega; Service-Apotheek Zuidhorn, Zuidhorn.

## Pre-publication history

The pre-publication history for this paper can be accessed here:

http://www.biomedcentral.com/1471-2393/14/166/prepub
